# Draft Genome Sequence of Manganese-Oxidizing Bacterium Massilia sp. Strain Mn16-1_5, Isolated from Serpentine Soil in Taitung, Taiwan

**DOI:** 10.1128/MRA.00694-19

**Published:** 2019-08-08

**Authors:** Sheng-Chung Chen, Chuen-Huey Chiu, Pin-Tzu Chiu, Yo-Chia Chen, Yong-Hong Lin, Chiu-Chung Young, Juei-Yu Chiu

**Affiliations:** a Department of Life Sciences, National Chung Hsing University, Taichung, Taiwan; b Department of Environmental Science and Engineering, National Pingtung University of Science and Technology, Pingtung, Taiwan; c Department of Biological Science and Technology, National Pingtung University of Science and Technology, Pingtung, Taiwan; d Department of Plant Industry, National Pingtung University of Science and Technology, Pingtung, Taiwan; e Department of Soil and Environmental Sciences, National Chung Hsing University, Taichung, Taiwan; University of Southern California

## Abstract

Massilia sp. strain Mn16-1_5 was isolated from serpentine soil. This strain is able to oxidize manganese and has the potential for bioremediation of chromium. Here, we present a 5.53-Mb draft genome sequence of this strain with a G+C content of 64.8% that might provide more information for species delineation and oxidase genes in this strain.

## ANNOUNCEMENT

The strain Mn16-1_5 was originally isolated from serpentine soil at Shitou Mountain (22°47′35.76″ N, 121°09′27.52″ E) in Taitung, Taiwan. Mn nodules (∼30 mg) collected from soil samples were immersed in a 5-ml sterile 0.1 M MnSO_4_ solution (pH 6.0) for 12 days. After centrifugation, the pellet was used for isolating Mn-oxidizing bacteria (MOB) by the spread plate method with a series of 10-fold dilutions onto the ATCC 279 broth agar plates which contained manganese. All plates were incubated at 26°C. MOB were identified by using Leucoberbelin blue (LBB) (1). Each of the single LBB-positive colonies was transferred for at least five rounds of streaking for purification, and purity was verified based on the morphology under phase-contrast microscopy (Carl Zeiss Axiolab). The purified strains were routinely cultured on ATCC 279 broth and preserved as glycerol stocks (20%, wt/vol) at −80°C for further study. One of the purified strains, designated Mn16-1_5, presented high manganese oxidation activity. Genomic DNA of strain Mn16-1_5 was extracted from colonies using the DNeasy plant kit (Qiagen). The 16S rRNA gene was amplified by using a universal bacterial primer set (2). Based on analysis of the 16S rRNA gene by BLASTN with default settings, strain Mn16-1_5 (GenBank accession number MK784972) belongs to the genus *Massilia* and is most closely related to type strain M. suwonensis 5414S-25 (98.56% sequence similarity).

For whole-genome shotgun sequencing, a single colony of strain Mn16-1_5 was transferred to 10 ml liquid ATCC 279 broth and incubated at 26°C and 150 rpm for 5 to 7 days. The total 200 ng of genomic DNA extracted by using the QIAamp genomic DNA kit (Qiagen) was fragmented using an S2 focused ultrasonicator (Covaris, Inc.). End-repaired DNA was further processed for ligation of Illumina adaptors with a TruSeq Nano DNA high-throughput library prep kit (96 samples) (Illumina, Inc.) and TruSeq DNA combinatorial dual (CD) indexes (96 indexes and 96 samples) (Illumina, Inc.). The adaptor-ligated enriched library was purified using sample purification beads (SPB). Library size distribution was checked on a 2100 Bioanalyzer high-sensitivity DNA chip (Agilent Technologies, Inc.). Genome sequencing was performed at the Genomics BioSci & Tech Co., Ltd. (Taipei, Taiwan) using the MiSeq platform (Illumina, Inc.) with a 2 × 300-bp paired-end read length sequencing protocol. A total of 2,402,818 reads with a Q30 cutoff were obtained by MiSeq sequencing. MultiQC v1.2 was used to evaluate the raw sequence data for quality control (3), followed by *de novo* assembly using SPAdes v3.10.1 (4). QUAST v.4.5 was used for quality assessment of genome assemblies (5). Default parameters were used in all of the bioinformatic analyses. The sequencing protocol generated 138× mean coverage of the genome. The draft genome of strain Mn16-1_5 comprised 88 contigs with an average G+C content of 64.8% and a total estimated size of 5,525,646 bp with an *N*_50_ value of 258,564 bp.

Gene annotations were performed by the NCBI Prokaryotic Genome Annotation Pipeline (PGAP) (6, 7). A total of 5,001 open reading frames (ORFs) were predicted, including 4,835 protein-coding sequences, 68 tRNAs, 23 rRNAs, and 4 noncoding RNAs (ncRNAs). The draft genome sequence of *Massilia* sp. strain Mn16-1_5 will be useful for understanding its phylogenetic taxonomy, metabolic activities, and biotechnological applications.

### Data availability.

This whole-genome shotgun project has been deposited at DDBJ/ENA/GenBank under the accession number PPXQ00000000. The version described in this paper is the first version, PPXQ01000000. The SRA accession number is PRJNA431474.
